# Bilateral Angle Narrowing and Acute Myopia Induced by Indapamide: A Case Report

**DOI:** 10.1155/2018/1486128

**Published:** 2018-12-02

**Authors:** Ana Catarina Pedrosa, Joana Rodrigues Araújo, João Paulo Macedo, Sérgio Estrela Silva, António Melo, Fernando Falcão-Reis

**Affiliations:** ^1^Department of Ophthalmology, Centro Hospitalar São João, Porto, Portugal; ^2^Department of Sense Organs, Faculty of Medicine of the University of Porto, Porto, Portugal

## Abstract

**Purpose:**

To describe a clinical case of indapamide induced bilateral angle narrowing and acute myopia.

**Materials and Methods:**

Clinical case report.

**Results:**

A 37-year-old Caucasian emmetropic man presented to the Emergency Department with complaints of acute-onset bilateral blurry vision, nine days after starting treatment for arterial hypertension with a combination of indapamide and amlodipine. Clinical examination revealed the presence of myopia and appositional closure of the anterior chamber angle. Ultrasound biomicroscopy and mode B ultrasonography disclosed bilateral ciliochoroidal effusion with anterior rotation of the ciliary body and iridocorneal angle narrowing. After intraocular pressure control with brimonidine and timolol, and replacement of indapamide/amlodipine by amlodipine only, the patient was discharged. Complete resolution of the clinical manifestations was observed after three weeks, with no sequelae.

**Conclusions:**

Indapamide may cause acute myopia and angle closure secondary to ciliochoroidal effusion that are fully reversible after drug withdrawal, as long as timely diagnosis is established. Therefore, indapamide, as well as other sulfonamide-derived drugs, must always be considered in the differential diagnosis of acute myopia and angle closure.

## 1. Introduction

Acute myopia and secondary angle closure are potential adverse effects of sulfa-containing medications [1–3]. Topiramate is the most frequently reported but numerous other drugs may be involved [1]. Although these adverse effects are described as rare [4], the causative drugs are commonly prescribed, and thus this entity may be more frequently encountered in clinical practice than one could expect. In this paper, we describe a case of acute myopia and secondary angle narrowing induced by indapamide, a sulfonamide-derived drug.

## 2. Materials and Methods

This is a clinical case report.

## 3. Results

A 37-year-old Caucasian man presented to the Emergency Department with complaints of acute-onset bilateral blurry vision. The symptoms had started two days before, and though distance vision was severely affected, near vision was preserved. The patient had never worn glasses or contact lenses and reported excellent previous uncorrected visual acuity in both eyes. He was overweight and had been recently diagnosed with arterial hypertension. Nine days before, he had started treatment with a combination of indapamide (1.5 mg) and amlodipine (5 mg). The medical history was otherwise unremarkable, regarding general and ocular health.

**Figure 1:**
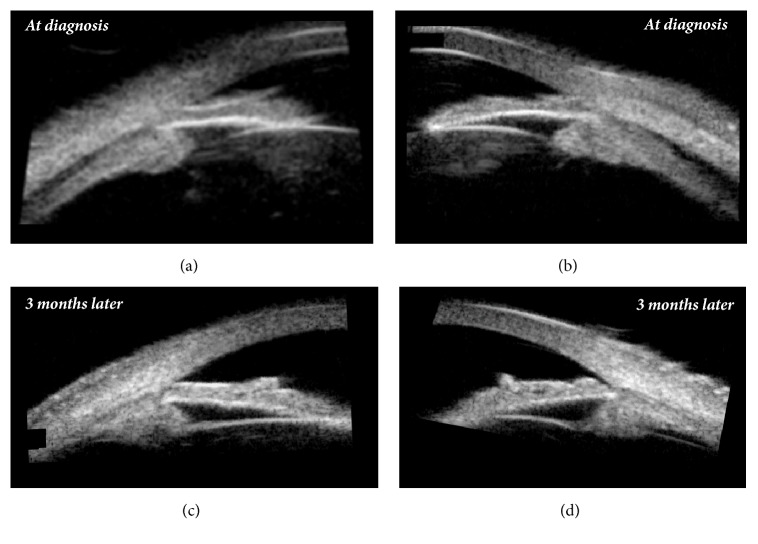
Ultrasound biomicroscopy showing, at diagnosis, supraciliary effusion, anterior rotation of the ciliary body and iridocorneal angle narrowing ((a) right eye; (b) left eye) and, three months later, resolution of the effusion and widening of the iridocorneal angle ((c) right eye; (d) left eye).

**Figure 2:**
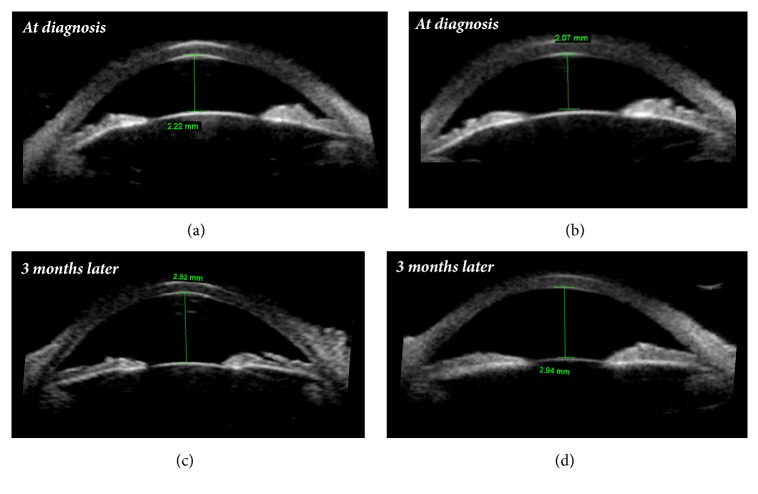
Ultrasound biomicroscopy showing shallow anterior chamber at diagnosis ((a) right eye, with anterior chamber depth of 2.22 mm; (b) left eye, with anterior chamber depth of 2.07 mm), and deepening of the anterior chamber three months later ((c) right eye, with anterior chamber depth of 2.92 mm; (d) left eye, with anterior chamber depth of 2.94 mm).

**Figure 3:**
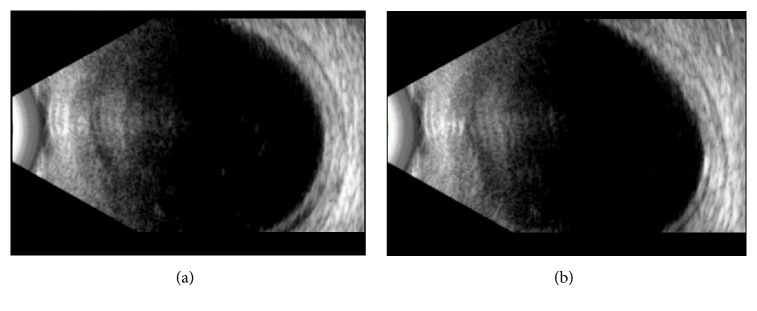
Mode B ultrasonography showing choroidal effusion at diagnosis ((a) right eye; (b) left eye).

Distance visual acuity was 20/100 unaided in both the right eye (OD) and left eye (OS). However, it increased bilaterally to 20/20 with correction of -2.75 -1.25x100 in OD and of -3.50 -0.75x80 in OS. Uncorrected near visual acuity was preserved, as the patient was able to read J1 when holding the Jaeger Eye Chart at a distance of 35 cm. Pupillary reflexes were normal. On slit-lamp examination, both eyes presented shallow anterior chambers, centrally and peripherally (grade 2, according to the Van Herick classification system). Intraocular pressure measured by Goldmann applanation tonometry was 36 mmHg in OD and 34 mmHg in OS. Gonioscopy revealed appositional closure of the anterior chamber angle (grade 2, according to the Shaffer classification system, and (A)B20b2+, according to the Spaeth classification system). Undilated fundus examination was unremarkable, with normal optic discs. Ultrasound biomicroscopy and mode B ultrasonography were performed. They disclosed bilateral ciliochoroidal effusion with anterior rotation of the ciliary body and iridocorneal angle narrowing (Figures 1–3).

After topical treatment with brimonidine, 2 mg/mL, and timolol, 5mg/mL, the intraocular pressure decreased to 24 mmHg in both eyes. Thus, the patient was discharged under treatment with those drugs, and indapamide/amlodipine (1.5 mg/ 5 mg) was replaced by amlodipine (10 mg) only.

The patient was then regularly monitored, and complete resolution of the symptoms was seen after three weeks. By then, distance visual acuity was 20/20 unaided in both eyes, and intraocular pressure was 16 mmHg in OD and 14 mmHg in OS without treatment. Ultrasound biomicroscopy confirmed resolution of the supraciliary effusion, with deepening of the anterior chamber and widening of the iridocorneal angle (Figures 1 and 2), and Humphrey automated perimetry showed no defects.

## 4. Conclusions

To date, there are four published cases of indapamide-induced acute myopia [3, 5–7], and only one of acute angle closure [6]. Our patient's complaints were mainly attributable to the myopic shift but objective examination showed that he also had moderate ocular hypertension secondary to appositional angle closure. The temporal relationship between the beginning of treatment with indapamide and the onset of symptoms, as well as the prompt resolution of all clinical findings after indapamide withdrawal, clearly indicates that indapamide was the causative agent.

In all cases reported to date, including ours, acute myopia and angle closure caused by indapamide were associated with ciliochoroidal effusion [3, 5, 6]. Although this is not definitively established, it is believed that ciliochoroidal effusion caused by indapamide and other sulfonamides represents an idiosyncratic reaction [1, 4]. Thus, it develops in rare susceptible patients, probably mediated by an abnormally increased synthesis of prostaglandins [2]. In fact, it has been shown that indapamide stimulates the synthesis of prostaglandin E2; in susceptible individuals, the presence of other contributing factors, such as an inborn error in eicosanoid metabolism or a clinical or subclinical infection, might lead to the local accumulation of prostaglandins during treatment with indapamide, causing vasodilation, increased vascular permeability, and ultimately ciliochoroidal effusion [2]. With ciliochoroidal effusion and anterior rotation of the ciliary body, the iris-lens diaphragm moves forward, the zonules relax, and the lens thickens, which results in myopic shift and narrowing or closure of the anterior chamber angle [1, 4].

According to the available evidence, indapamide-induced acute myopia and angle closure appear to have an excellent prognosis, as long as timely diagnosis is established [3–6]. However, failure to identify indapamide as the etiological factor might prevent adequate treatment and lead to irreversible optic nerve damage in cases of angle closure. Iridotomy is not effective as the pathogenic mechanism is not pupillary block [3, 5]. Therefore, sulfonamide-derived drugs such as indapamide must always be considered in the differential diagnosis of acute myopia and angle closure.
